# Using Mobile Technology (pMOTAR) to Assess Reactogenicity: Protocol for a Pilot Randomized Controlled Trial

**DOI:** 10.2196/resprot.9396

**Published:** 2018-10-03

**Authors:** Kathryn Therese Mngadi, Bhavna Maharaj, Yajna Duki, Douglas Grove, Jessica Andriesen

**Affiliations:** 1 Centre for the AIDS Programme of Research in South Africa Durban South Africa; 2 Clinical Research Department The Aurum Institute Johannesburg South Africa; 3 Fred Hutchinson Cancer Research Center Seattle, WA United States

**Keywords:** research protocol, mobile health application, HIV preventive vaccines, telemedicine, mobile applications, AIDS vaccines

## Abstract

**Background:**

Accurate safety monitoring in HIV vaccine trials is vital to eventual licensure and consequent uptake of products. Current practice in preventive vaccine trials, under the HIV Vaccine Trials Network (HVTN), is to capture related side effects in a hardcopy tool. The reconciliation of this tool, 2 weeks after vaccination at the safety visit, is time consuming, laborious, and fraught with error. Unstructured Supplementary Service Data (USSD), commonly used to purchase airtime, has been suggested for collection of safety data in vaccine trials. With saturated access to mobile phones in South Africa, this cheap, accessible tool may improve accuracy and completeness of collected data and prove feasible and acceptable over the hardcopy tool.

**Objective:**

The objective of our study is to develop and implement a USSD tool for real-time safety data collection that is feasible and acceptable to participants and staff, allowing for a comparison with the hardcopy tool in terms of completeness and accuracy.

**Methods:**

This feasibility study is being conducted at a single study site, the Centre for the AIDS Programme of Research in South Africa eThekwini Clinical Research site, in South Africa. The feasibility study is nested within a parent phase 1/2a preventive HIV vaccine trial (HVTN 108) as an open-label, randomized controlled trial, open to all consenting parent trial participants. Participants are randomly assigned in a 1:1 ratio to the hardcopy or USSD tool, with data collection targeted to the third and fourth injection time points in the parent trial. Online feasibility and acceptability surveys will be completed by staff and participants at the safety visit. We will itemize and compare error rates between the hardcopy tool and the USSD printout and associated source documentation. We hypothesize that the USSD tool will be shown to be feasible and acceptable to staff and participants and to have superior quality and completion rates to the hardcopy tool.

**Results:**

The study has received regulatory approval. We have designed and developed the USSD tool to include all the data fields required for reactogenicity reporting. Online feasibility and accessibility surveys in both English and isiZulu have been successfully installed on a tablet. Data collection is complete, but analysis is pending.

**Conclusions:**

Several HIV preventive vaccine trials are active in Southern Africa, making tools to improve efficiencies and minimize error necessary. Our results will help to determine whether the USSD tool can be used in future vaccine studies and can eventually be rolled out.

**Trial Registration:**

ClincalTrials.gov NCT02915016; https://clinicaltrials.gov/ct2/show/NCT02915016 (Archived by WebCite at http://www.webcitation.org/71h0cztDM)

**Registered Report Identifier:**

RR1-10.2196/9396

## Introduction

### Rationale

Despite the gains made in the provision of antiretroviral treatment and, more recently, prevention globally, in 2015 there were 1.8 million new infections worldwide. South Africa has the biggest antiretroviral treatment program in the world, with approximately 3.4 million people being treated [1]. In November 2015, South Africa registered emtricitabine/tenofovir disoproxil fumarate (Truvada) for preexposure prophylaxis in key populations yet, in 2016 alone, approximately 270,000 incident infections occurred [2]. A safe, effective HIV vaccine is still seen as the solution to epidemic control and, ultimately, elimination.

Safety monitoring in HIV vaccine clinical trials is vital, from early-phase safety and immunogenicity testing, to late-phase efficacy testing and eventual licensure of candidate vaccines. An essential part of safety monitoring is the collection of side effects data reported by participants after they have received a study product and for 3 to 7 days after leaving the clinic. All preventive HIV vaccine trials collect reactogenicity data: this is a set of known and expected injection site and systemic symptoms and signs that are related to the vaccine. Unexpected side effects (classified as adverse events) are also collected on study-specific case report forms (CRFs). The duration of the reactogenicity period depends on the protocol, and adverse event data collection periods are defined per protocol, depending on the seriousness of the event. In sub–Saharan Africa, a suite of trials in response to the modest results of the RV144 trial [3] has been launched to progress a modified HIV clade C–specific vaccine candidate to licensure and to deepen the understanding of the mechanisms of immune protection against HIV. Concurrently, several early-phase HIV vaccine trials, 2 efficacy trials, and a proof-of-concept neutralizing antibody infusion trial are being conducted. The possibility of increased occurrence of safety events is thus greater in these populations and simultaneously demands more accurate, efficient methods of data collection.

### Vaccine Uptake and Efficacy

Public perception and tolerance of licensed vaccine risk indicates that, in the absence of a direct threat from disease, some people will not undergo vaccination unless absolute safety can be assured [4]. If a successful HIV vaccine candidate is licensed in the future, there is a risk that people may avoid vaccination due to safety concerns. A systematic review of barriers to participating in an HIV vaccine trial analyzed common themes between studies and found that vaccine side effects and safety were noted as barriers to participation [5]. The collection of accurate safety data is vital to address these concerns. In a systematic review of 50 randomized controlled vaccine trials in developing countries, consistent documentation was key to the successful implementation of international safety standards in resource-poor settings [6]. Modern technologies, including short message service (SMS) and mobile phone apps, were recommended as possibly facilitating the monitoring of vaccine safety in remote areas, where access to internet connectivity may not always be possible [6]. This technology would allow for real-time data collection, offering an improvement over the hardcopy tool. Standardization of safety reporting across multiple sites in developing countries was borne out in a systematic review of safety data reporting in vaccine trials for malaria, tuberculosis, and HIV, which focused partly on methods used to collect and report side effects [7]. This review noted imprecision and inconsistency of body temperature reporting, which is a key objective safety parameter. The proper collection and documentation of unexpected side effects also allows for regulators and sponsors alike to link uncommon side effects across trials and sites, enabling the identification of sporadic, serious side effects [7].

### The Hardcopy Tool

Research staff collect reactogenicity data initially at the study site before and up to 60 minutes after vaccination on CRFs. For the remainder of the reporting period, participants collect data off-site on a hardcopy tool. This demands extensive, intensive training of participants on completion of this tool (in the language of their choice) and training on the use of a thermometer and ruler, for temperature and injection site reactogenicity assessments, respectively. Participants are also taught to objectively grade the severity of symptoms using a standardized set of symptom criteria. Research staff are directed to contact participants by telephone daily (in early-phase protocols) or after 3 or 7 days (for later-phase protocols) to check on participants’ health, collect and objectively grade symptoms, and provide refresher training as needed. Objective grading determines whether a symptom is mild, moderate, severe, or life-threatening and facilitates clinical decision making and symptom management on continuation of vaccinations per participant and protocolwide. Staff document these data directly onto corresponding CRFs on working days and in a site-developed document outside of clinic operational hours. This is then transcribed to CRFs on the next working day. At the 2-week postvaccination safety visit, research staff review the CRFs, hardcopy tool, and site-developed source document (if applicable) with each participant to ensure that CRF data are accurate. This process is repeated for each vaccination visit within the protocol.

This exercise remains fraught with error from a multitude of sources: daily data collection is not directly attributable to participants, and there is no evidence that data are completed contemporaneously; participants are often uncontactable at the agreed-upon time or do not have the hardcopy tool with them when contacted by staff to complete CRFs; participants may not return the hardcopy tool to the site at the safety visit or at all, and data have to be reconstructed based on participant recall 2 weeks later; participants tend to grade symptoms subjectively despite training; many hardcopy tool entries are incomplete or incorrect; and staff need to document each error in detail between the relevant documentation, making the time and labor the task consumes proportional to the number of errors. In addition, national research regulators may consider all or part of the hardcopy tool as a source document, and errors such as incorrect dates, overwriting, and entry of redundant or unclear information by the participant must be documented in explicit detail. If not, the site risks incurring serious findings by external monitors, directly affecting protocol quality metrics. For moderate reactogenicity and other adverse events, the site relies on participant-initiated contact to determine whether a clinic visit is required as mandated by the protocol. If this is missed, a protocol deviation must be reported to the sponsor, regulator, and ethics committee. More importantly, participant safety may be severely compromised if an unreported symptom fulfills a pause rule for the study, when all further vaccinations at all sites may be held, pending a risk assessment.

The alternative to the systems detailed above would be daily clinic visits for the duration of the reactogenicity period for each vaccination time point. This would increase the cost of participant reimbursements, negatively affect travel and convenience for participants, and increase the research site’s workload for the day. The conduct of multiple studies per site, necessitating a process efficiency system to reduce visit duration, does not support this labor-intensive and inefficient process.

### Mobile Health and Unstructured Supplementary Service Data

Mobile health (mHealth) is the practice of medicine and public health supported by mobile devices, which have the potential to facilitate alerts, reminders, and data collection, substantially reducing the burden on health care systems [8-11]. Unstructured Supplementary Service Data (USSD) is a tool that transfers messages directly over the mobile operator network, allowing for an exchange between mobile phones and a network app. It is accessed by user request, making use of short codes or text strings to trigger certain services and facilitate high-speed, interactive, session-based communication. The text string, up to 160 characters long, can be used to establish a new session or to continue an established session, with asterisk (*) and hash (#) codes signifying the beginning and end of the request, respectively. Most importantly, it is accessible on basic mobile phones. In Botswana, research recommendations in mHealth have alluded to the successful use of USSD by health care workers to retrieve treatment guidelines [12].

South Africa is highly ranked fifth in the world for mobile data usage [13,14], with more active subscriber identity module cards than people and 128% active mobile connections among the population [15]. By mid-2013, total mobile phone subscriptions were estimated at over 68 million [13]. USSD could prove useful to implement real-time reactogenicity data collection, having several advantages courtesy of its menu-based platform, namely, speed and responsivity, affordability, real-time entry and access, automation, user initiation, and simultaneous mass usage. It is affordable and accessible, with the total cost of a typical session depending on duration of the session (20 cents per 20 seconds; US $0.074 dollar = 100 cents or 1 R). It allows only “yes” or “no” responses, thus shortening the session length and improving affordability. SMS text messaging is managed manually, can cost up to 100 cents per message, and would need to be initiated by the provider. Responses submitted by USSD are automatically deleted on the device, complying with data confidentiality requirements of Good Clinical Practice and the US Food and Drug Administration’s Code of Federal Regulations Part 11. One of the cornerstones of the South African National Department of Health’s mHealth strategy [16] commits the government to providing an mHealth implementation plan to strengthen research and development.

In this study, we propose the collection of reactogenicity and adverse event data using a customized USSD tool, with the objectives of developing and implementing a basic mobile phone-based USSD tool for the collection of reactogenicity data, establishing feasibility and acceptability among research staff and participants, and determining whether the accuracy and completeness is superior to the hardcopy tool. Piloting this technology in the research setting in a developing country such as South Africa in an early-phase trial will allow for optimization of the system to facilitate collection of safety data across multiple sites, in late-phase, large-scale studies and eventual programmatic rollout.

The South African regulator follows *South African Good Clinical Practice Guidelines* [17], which confirms the compliance of USSD data collection as acceptable.

## Methods

### Study Design

This is an open-label, randomized controlled trial under the HIV Vaccine Trials Network (HVTN) nested in the parent HVTN 108 trial, a phase 1/2a clinical trial to evaluate the safety and immunogenicity of HIV clade C DNA, and of MF59- or AS01B-adjuvanted clade C Env protein in various combinations, in healthy, HIV-uninfected adult participants. The parent study has a series of 4 vaccination time points in a 6-month period: at months 0, 1, 3, and 6. The study will use 2 consecutive vaccination time points, namely months 3 and 6, to implement the USSD tool, with postvaccination feasibility and acceptability surveys at the 2-week postvaccination safety visits. At months 0 and 1, all participants complete the hardcopy tool for reactogenicity assessment. The intervention is a purpose-designed, study-specific USSD tool that collects all the protocol-mandated reactogenicity data collected by the hardcopy tool. At the time of initiating this paper, the USSD tool and electronic surveys had been developed and approved, and enrollment was ongoing.

### Setting

The Centre for the AIDS Programme of Research in South Africa (CAPRISA) eThekwini Clinical Research Site (ECRS) was chosen as the sole site for this study.

### Approvals

The parent and this study were approved by the University of Kwazulu-Natal’s Biomedical Research Ethics Committee; in addition, the parent study was approved by the University of Kwazulu-Natal’s Institutional Biosafety Committee, the South African Medicines Control Council, and the Department of Agriculture, Forestry and Fisheries. The South African National Clinical Trials Registry number for the parent study is NCT02915016. This pilot trial did not meet the US Food and Drug Administration requirements for trial registration. We received further review and approvals for this study from the HVTN 108 protocol team, the HVTN Regulatory Affairs office, HVTN Initiatives Program’s review board, and HVTN Scientific Governance Committee.

### Statistical Considerations

#### Accrual

Up to 30 slots may be allocated to the CAPRISA ECRS for the parent study. Recruitment for this nested study will target enrolling all consenting, healthy, HIV-uninfected adult participants aged 18 to 40 years enrolled in the parent study at the CAPRISA ECRS in a 1:1 ratio to the hardcopy or USSD tool.

#### Sample Size Calculations

We estimated the power for detecting a reduction in error rates for the USSD relative to the hardcopy tool arm via simulation. We assumed that the total number of errors for a single participant at a single visit would follow a Poisson distribution whose mean we determined by the study arm the participant was assigned to and differs depending on whether the participant was randomly assigned to use the USSD or the hardcopy tool. Data were simulated assuming 2 possible error rates for the hardcopy group and 2 possible group sizes, and the error rate for the USSD group was varied over a small range of values. The simulated data were fit using a Poisson regression model consisting of an intercept and a term for the study group, and the coefficient of the study group was tested for equality with 0.

#### Study End Points

We will calculate error rates for both the USSD and hardcopy tool for completeness (defined as an entry that should have been completed, but was not) and for accuracy (the level of agreement between the data collected either by hardcopy tool or by USSD and the entries on the reactogenicity CRFs following a discussion with the participant to confirm final data; staff transcription errors and participant completion errors will also be taken into account). This will be expressed as errors per 100 pages completed.

#### Conditions Under Which Power is Computed

For means, the reported error count range for the hardcopy tool is 5 (“very good”) to 26 (“very bad”). We used a calculated average (mean) error rate, likely lying in the range of 13 to 17, to compute power (Table 1).

For sample size, we used a maximum possible enrollment into this ancillary study of 30 participants, based on the expected slot allocation for HVTN 108, which would result in 15 participants assigned to each of the USSD and hardcopy arms. Since we are unlikely to achieve the maximum enrollment, we computed power for 2 levels of enrollment that reflect poor and good consent rates. The poor level assumes that a total of 20 participants consent to the ancillary study—an expected 10 per group—which is a 56% consent rate. The good level of enrollment assumes that 30 participants will consent—an 83% rate—and would give us an expected 15 participants per group.

Since the data consist of repeated measures from individual participants, they require modelling that accounts for the correlation between measurements from the same person, and one standard way is to use mixed-effects models. Count data require us to use the generalized linear mixed-effects model framework, and we will employ the negative binomial family (rather than the Poisson) for the extra flexibility it provides. Our model will contain at a minimum a fixed effect for the method of data collection (USSD or hardcopy tool) and a random effect for participant. When writing the statistical analysis plan, we may consider models containing additional fixed-effect terms (eg, for sex, visit number), as well as more complex random-effect structures, and we will incorporate terms as appropriate based on model fit criteria such as the Akaike information criterion. Inference on the difference between methods will be made by testing whether the estimate of the collection method parameter is significantly different from 0 at the .05 level.

**Table 1:** Power for differential sample size and error rates.

Mean errors per participant per visit		Reduction in error rate^a^, n (%)	Power to detect difference in means between HCT^b^ and USSD^c^ groups (%)	
HCT	USSD		10 participants per group	15 participants per group
17	14	3 (18)	40	55
17	13	4 (24)	65	80
17	12	5 (29)	83	94
17	11	6 (35)	95	99
13	10	3 (23)	51	69
13	9	4 (31)	80	90
13	8	5 (38)	95	99

^a^Unstructured Supplementary Service Data vs hardcopy too.

^b^HCT: hardcopy tool.

^c^USSD: Unstructured Supplementary Service Data.

#### Discontinuation and Early Study Termination

The number and percentage of participants who discontinue vaccination and thus who terminate the nested study early will be tabulated by reason and intervention arm in the pilot study.

#### Data Management

For participants enrolled into the hardcopy tool arm, the tool, CRFs, and other study documentation are the source for the reactogenicity data over the 7-day postvaccination reporting period.

For those randomly assigned to the USSD arm, the tool database printouts, CRFs, and other study documentation are the source for reactogenicity data. All participants randomly assigned to the USSD arm will also receive a backup hardcopy tool in the event of system errors that cannot be overcome with site support; these will also be used as source, if applicable. The tool database printouts of reactogenicity reports are transcribed onto CRFs by research staff.

### Tools

#### Unstructured Supplementary Service Data Tool Development

The tool has been designed through a collaborative effort between the service provider Channel Mobile (Cape Town, South Africa) in consultation with the investigators and the parent study HVTN Clinical Safety Specialist team. The tool name is pMOTAR (pilot study of Mobile Technology to Assess Reactogenicity) for ease of reference for participants and research staff.

The following design features facilitate data input and collection. (1) All the data elements are included in the hardcopy tool of the parent study. (2) As data are entered on the mobile phone, uploads to the database are immediate. (3) Participants can access the system multiple times in one day, to facilitate completion of incomplete sessions. (4) A “preserve state” enables participants to continue from the last active screen where they left off, if they were previously timed out or could not complete the session for any reason. (5) Only minimal responses are required for ease of use, for example, selection of a number corresponding to the symptom, followed by selection of a number corresponding to the objective grading. (6) Participant responses indicative of potential safety events, such as a “yes” response to any expected or unexpected symptoms followed by free text to denote the symptom, will also be available to staff through contemporaneous alerts from the system routed to designated staff mobile phone numbers. (7) Participants can choose between 2 languages: English and Zulu. (8) Risk of harm is minimal, as confidentiality is assured in that entered data cannot be saved to the handset or transferred to another handset, and are automatically deleted following submission; only research staff have access to the entered data in the tool database. (9) Reverse billing is applied, such that participants can log on to the system even if they have no airtime. At the end of the session, the cost of the session is charged to the research site. This ensures ease of access to and use of pMOTAR with no possibility of the participant running out of airtime during a session. (10) Automated SMS reminders are sent out at 08:00, 12:00, and 15:00 if the system is not accessed and the tool is not completed. (11) Double entries have not been disallowed so as to facilitate entry of updated measurements or missed entries from a previous day that have been captured on a hardcopy tool, perhaps because of connectivity issues on the day of measurement. Each entry will trigger an SMS text message to alert study staff, who can call the participant immediately to clarify the reason and document the same in a chart note, such that duplicate entries in the database can be explained and analyzed appropriately.

#### Unstructured Supplementary Service Data Tool Database

The following tool database features facilitate contemporaneous access of information by research staff. (1) Printouts from the tool database will serve as source documentation for completion of CRFs. (2) Real-time access to the tool database allows staff to review data in a timely manner and determine whether immediate action to assess a participant’s response is required to clarify an entry or to facilitate a site visit to assess safety. (3) Reports can be downloaded and printed, and usage can be tracked based on mobile phone number patterns from the tool database. (4) An audit trail and an automatic daily backup of the tool database is made to an external drive at 04:00. (5) Physical servers are hosted at a secure data center with failovers (switching to a redundant or standby computer server on the failure or abnormal termination of the current server) to the Amazon cloud (Amazon.com, Inc, Seattle, WA, USA) and Azure (Microsoft Corporation, Redmond, WA, USA).

#### Unstructured Supplementary Service Data Tool Specifics

**Figure 1:**
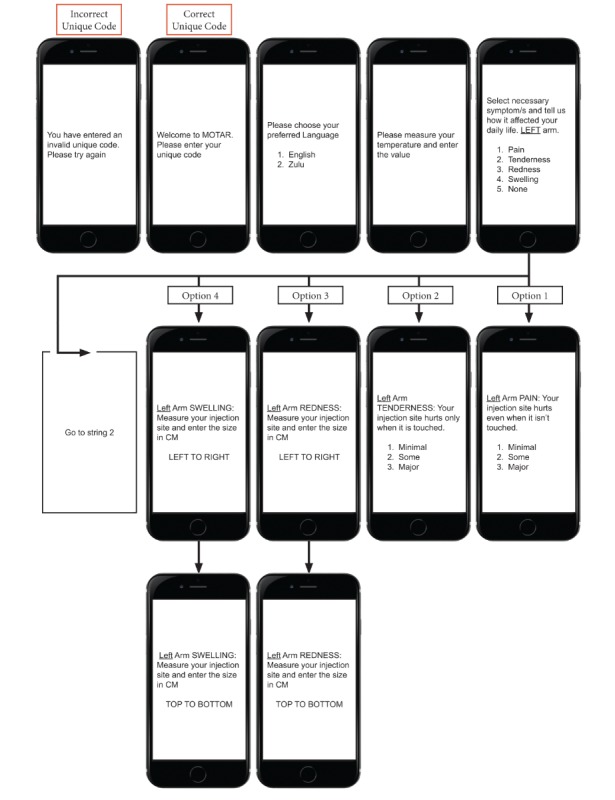
The Sample Unstructured Supplementary Service Data Tool (string 1).

**Figure 2:**
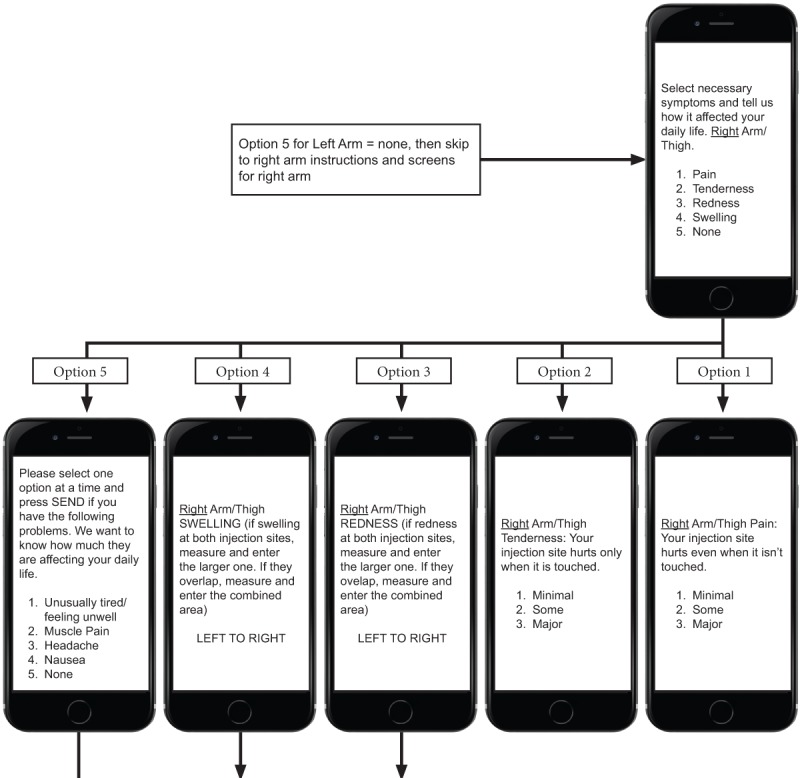
Part 1 of the Sample Unstructured Supplementary Service Data Tool (string 2).

**Figure 3:**
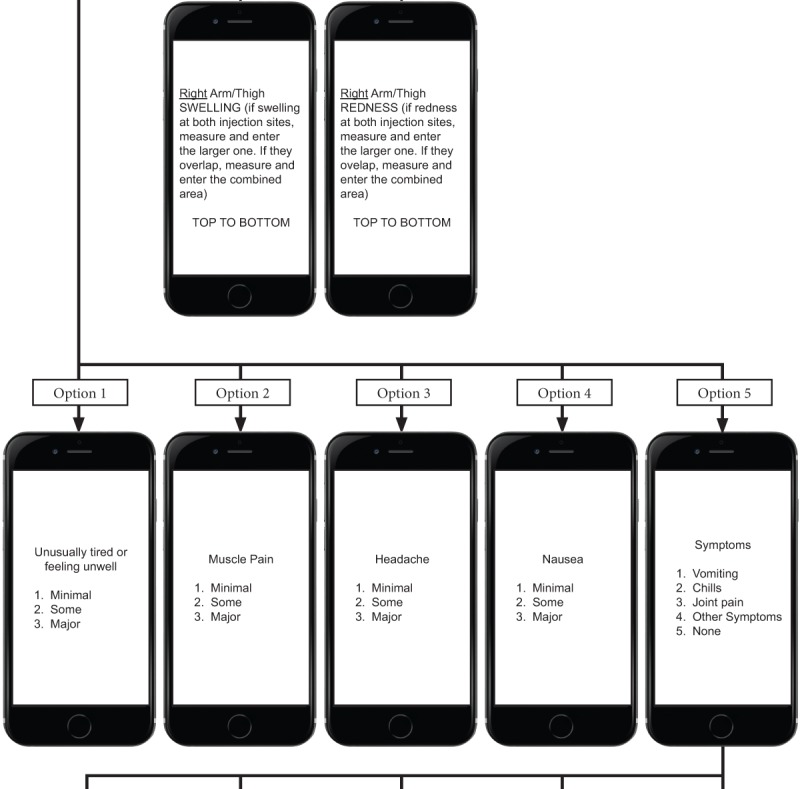
Part 2 of the Sample Unstructured Supplementary Service Data Tool (string 2).

**Figure 4:**
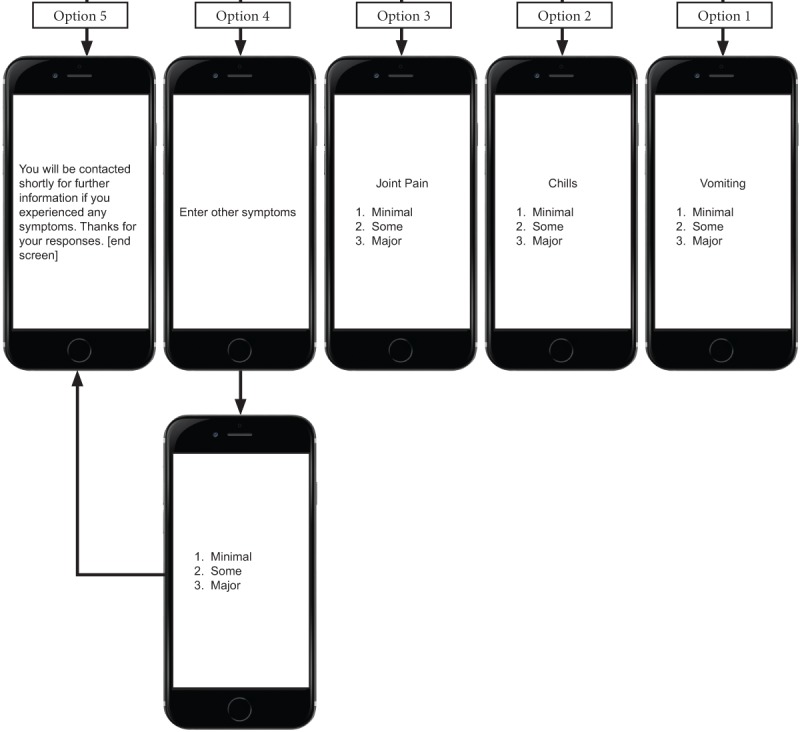
Part 3 of the Sample Unstructured Supplementary Service Data Tool (string 2).

The flow of the USSD app service for reactogenicity symptom assessment is as follows. First, a mobile user initiates the service by dialing the USSD string defined by the service provider Figures 1-4 show sample screen flow strings.

Second, the USSD app server receives the service request from the user and responds by sending the user a menu of options. (1) The first menu allows selection of the preferred language, either 1 for English or 2 for Zulu; the user responds by selecting option 1 or 2 and then presses <SEND>. If the incorrect option is selected, the user selects <CANCEL> and reselects. (2) The next screen includes a greeting and a request to enter a 4-digit unique identifying code provided by the research staff. (3) Once the code is entered, an invitation to capture the temperature is loaded, allowing the participant to enter a measurement to 1 decimal point. (4) After the participant selects <SEND>, an automated menu of numbered *local* symptoms and choice of responses is sent to the user. Local symptoms also reflect whether vaccinations are given in the right or left upper arm, or both. (5) If the participant selects either redness or swelling at the injection site, they will be prompted to enter measurements in centimeters, initially from top to bottom, and then from side to side. (6) If the participant responds by making a single selection of another symptom, this will trigger an automated response from the app, which sends out the selection of grading (minimal, some, or major) by the participant. (7) Once the participant selects <SEND>, they are returned to the original symptom selection screen to select any other symptoms that may be experienced. (8) If no other symptoms have been experienced, the participant selects <NEXT>, then the system loads the selection of *systemic* symptoms for completion. (9) If the participant responds by making a single selection of a symptom, this will trigger an automated response from the app, which sends out the selection of grading (minimal, some, or major, which equates to minimal, moderate, and severe) by the participant. (10) Once the grading for the selected symptom is selected and the participant selects <SEND>, they will be taken back to the original systemic symptom selection screen. 

(11) The last prompt from the system is for the occurrence of any other unexpected symptoms, not previously listed on any screens, and allows for free entry of text to describe the symptom. (12) Each response occurs in a matter of seconds, in quick succession, and in real time, and the participant is not able to skip any screens to get to the end.

Third, once a participant selects <SEND>, the entered data are automatically uploaded to the tool database and simultaneously deleted from the handset, ensuring confidentiality.

Fourth, the app automatically ends the session when <FINISH> is selected, then delivers a “Thank you” message to confirm completion of all questions to the participant.

Mobile phone numbers of consenting participants are obtained from the locator information of the parent study records and linked to unique confidential identifying 4-digit codes, which are assigned to the participant during training on the USSD program and used by the participant to access the app.

#### The Hardcopy Tool

**Figure 5:**
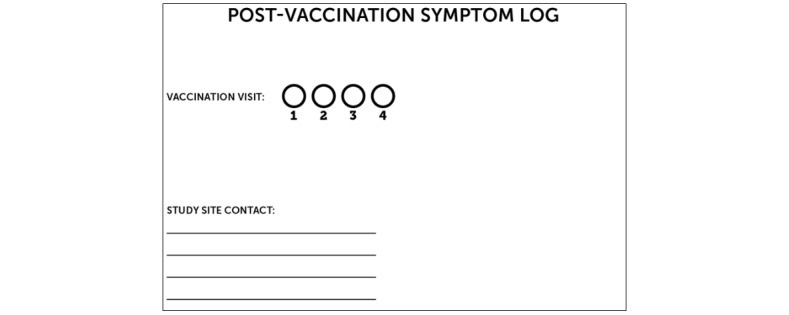
Sample hardcopy tool (page 1).

**Figure 6:**
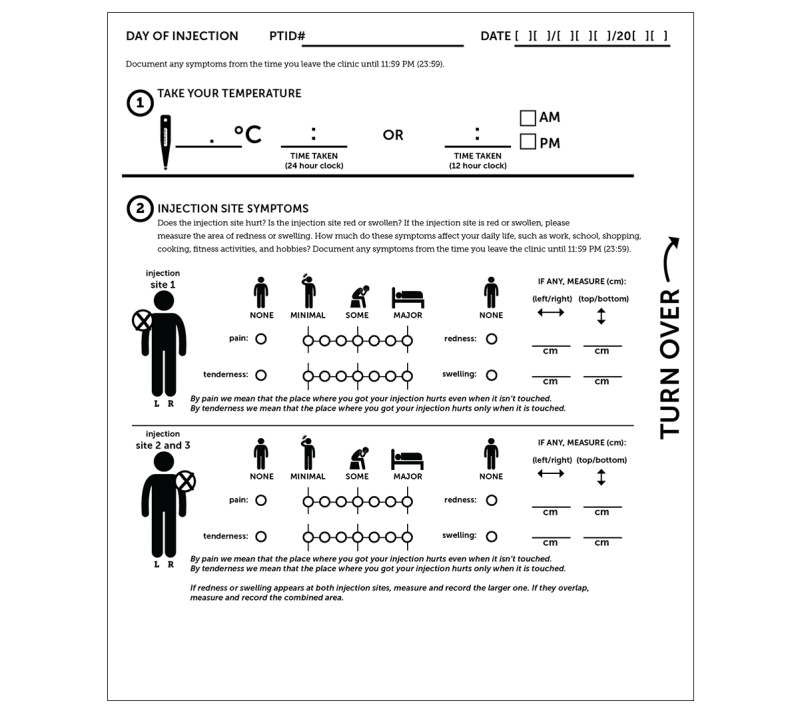
Sample hardcopy tool (page 2).

**Figure 7:**
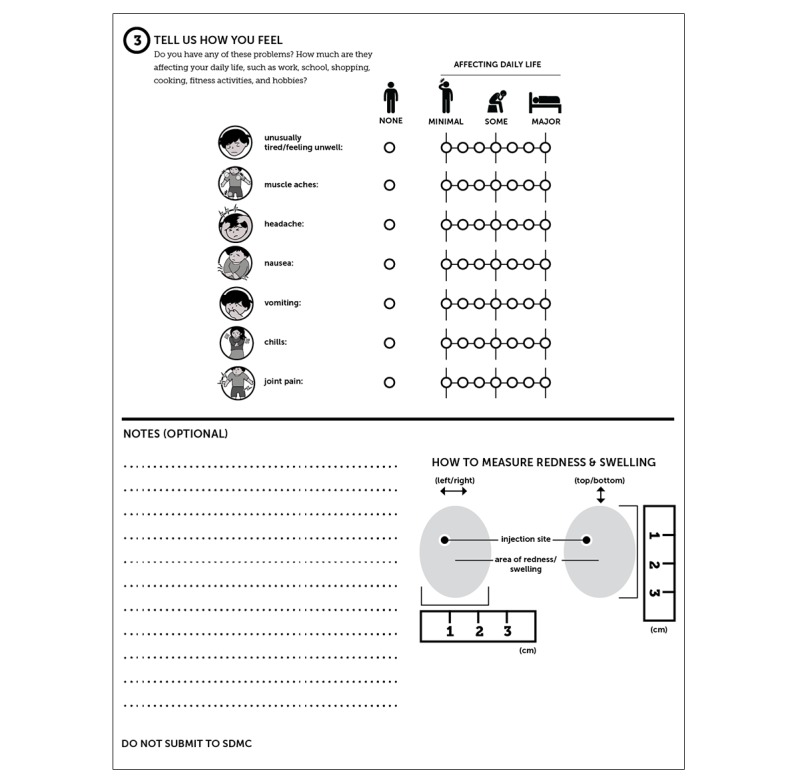
Sample hardcopy tool (page 3).

The hardcopy tool (Figures 5-7) is provided by the research staff to the participant, who is trained in how to complete this tool on the day of vaccination, before leaving the clinic. If a participant experiences difficulty completing the tool at home, they are informed to contact the site for support or inform the staff during the daily call. Research staff telephone participants either daily or according to the protocol directive, with the expectation that the participant has access to the hardcopy tool at the time of the call. Research staff will ask the participant to share temperature and any local injection site lesion measurements with the research staff member, who then enters this information either directly onto the CRF on a working day or into a site-developed document for use on nonworking days off-site.

### pMOTAR Clinical Procedures

#### Informed Consent and Screening Procedures

Research staff obtain written informed consent in the preferred language of the participant and prior to any screening procedures.

Participants are screened at a visit prior to the third vaccination in the parent study vaccination series, followed by an assessment of eligibility, as per the inclusion and exclusion criteria. Selected information from the parent study, namely informed consent form and locator information, are accessed to confirm eligibility. The participant identifying number from the parent study is used to link data from this ancillary study to the parent study.

#### Enrollment and Randomization

Participants are enrolled and randomly assigned on the same day as the third vaccination, following a review and confirmation of eligibility, revisiting the informed consent form (as needed). Participants in the intervention arm are assigned a unique 4-digit code to access the USSD tool; participants in the control arm follow procedures for the parent study.

The randomization system uses computer-generated random numbers, so that if 30 participants are enrolled, there will be 15 in each arm. Sealed opaque randomization envelopes are provided to the study coordinator for storage, to be opened in sequential order. After the envelopes are opened, the date and time of opening the envelopes, as well as the research staff member’s name, are documented on the envelope. This information is then noted on the randomization sheet.

All participants are trained in the use of either the hardcopy or USSD tool, depending on arm assignment, and in the use of the thermometer and ruler. The participants in the intervention arm are trained in the use of the hardcopy tool as a backup; this training is repeated at the next vaccination time point (3 months after enrollment). Likewise, all participants, irrespective of arm assignment, receive site contact details to assist with tool completion (if they experience difficulty once off-site) or to report additional problems.

#### Eligibility Criteria

Participants are healthy, HIV-uninfected (seronegative) adults enrolled in the parent study, who comprehend the purpose of the study and have provided written informed consent.

The inclusion criteria are (1) HIV-uninfected male and female adults, 18 to 40 years of age, who are enrolled in the parent study at the CAPRISA ECRS; (2) participants who have confirmed full access to a compatible mobile phone and willing to receive text reminders; (3) participants who have the ability and willingness to provide informed consent; (4) participants who are English or Zulu speaking; and (5) participants who have demonstrable text message literacy.

The exclusion criteria are (1) participants who have missed vaccination visits at month 0 or 1 in the parent study; (2) participants who have had vaccination visits discontinued (temporarily or permanently); (3) participants who have mobile phones on a contractual basis, paying a monthly service and airtime fee over 24 months from purchase, as reverse billing is not compatible with this system; and (4) participants who have any significant condition or process that renders the participant incapable of participating that would interfere with or serve as a contraindication to protocol adherence, assessment of safety or reactogenicity, or a volunteer’s ability to give informed consent as per the investigator’s decision.

#### Blinding

Participants and site staff are unblinded as to participant intervention group assignments for the 2 arms of this study but remain blinded to the treatment assignment of the parent study. The Statistical Data Management Centre staff for the parent study are blinded to the group assignments in this nested study to maintain the integrity of the blinding for the parent study.

#### Follow-Up

Follow-up visits are aligned with the 2-week postvaccination safety visit of the parent study at each designated vaccination time point. At these scheduled safety visits, in addition to the parent study procedures, a computerized feasibility and acceptability survey is completed on a tablet, by both participants and research staff members. Participants have separate surveys for either the USSD tool (Multimedia Appendix 1) or hardcopy tool (Multimedia Appendix 2), while staff have one survey that compares the two (Multimedia Appendix 3).

#### Reactogenicity Assessments

The reactogenicity assessment period for the parent study is 7 full days following each vaccination (Tables 2 and 3). For participants assigned to the hardcopy tool arm, participants complete the tool off-site daily for 8 days (day 0 to day 7, day 0 being the night of the vaccination). Research staff contact participants daily for the first 4 days only (a phone call is made to check on the reactogenicity assessment done the day before) during the assessment period to determine whether the objective grading, as per the US National Institute of Allergy and Infectious Diseases Division of AIDS (DAIDS) adult and pediatric toxicity table [18], is confirmed. If the grading is confirmed, a repeat call the next day will be made to determine whether the symptom is resolving. If the symptom is not resolving, the participant will be brought into the clinic for a clinician assessment within 48 hours of onset of the symptom, as per the protocol. Participants document day 4 to 7 data on the tool without daily telephone review by the staff and return the tool at the 2-week postvaccination safety visit for review and reconciliation with CRFs and other site documentation. Data from the day 0 to day 3 contacts are entered directly onto the relevant CRFs during working days and into site-developed source on nonworking days. Participants are instructed to alert the site of any moderate reactogenicity symptoms on the day or any unexpected adverse events, that is, symptoms not listed in the hardcopy tool.

**Table 2:** Reactogenicity procedures hardcopy versus Unstructured Supplementary Service Data (USSD) tool.

Reactogenicity day	Hardcopy tool	USSD tool
Day 0	Train on use of tool, thermometer, and ruler	Train on use of tool, thermometer, and ruler; provide unique access code
Day 1	Call participant and complete source documentation for reactogenicity for day 0; enter into parent study database	Send reminder text Access database and print reactogenicity data for day 0; transcribe onto CRF^a^and enter into parent study database
Day 2	Call participant and complete source documentation for reactogenicity for day 1; enter into parent study database	Send reminder to access the USSD Access database and print reactogenicity data for day 1; transcribe onto CRF and enter into parent study database
Day 3	Call and complete source documentation for reactogenicity for day 2; enter into parent study database	Send reminder mail to access the USSD by bulk SMS^b^text messaging Access database and print reactogenicity data for day 2; transcribe onto CRF and enter into parent study database
Day 4	Call and complete source documentation for reactogenicity for day 3; enter into parent study database	Send reminder to access the USSD Access database and print reactogenicity data for day 3; transcribe onto CRF and enter into parent study database
Day 5	Call and complete source documentation for reactogenicity for day 4; enter into parent study database	Send reminder to access the USSD Access database and print reactogenicity data for day 4; transcribe onto CRF and enter into parent study database
Day 6	Call and complete source documentation for reactogenicity for day 5; enter into parent study database	Send reminder to access the USSD Access database and print reactogenicity data for day 5; transcribe onto CRF and enter into parent study database
Day 7	Call and complete source documentation for reactogenicity for day 6; enter into parent study database	Send reminder to access the USSD Access database and print reactogenicity data for day 6; transcribe onto CRF and enter into parent study database
Day 8	Call and complete source documentation for reactogenicity for day 7; enter into parent study database	Send reminder to access the USSD Access database and print reactogenicity data for day 7; transcribe onto CRF and enter into parent study database

^a^CRF: case report form.

^b^SMS: short message service.

**Table 3:** Schedule of reactogenicity assessments.

Day	Time	Performed by
0^a^	Baseline: before vaccination	HIV Vaccine Trials Network clinical research site staff
0	Early: 25-60 minutes after vaccination	HIV Vaccine Trials Network clinical research site staff
0	Between early assessment and 11:59 PM	Unstructured Supplementary Service Data or hardcopy tool
1	Between 12:00 AM and 11:59 PM	Unstructured Supplementary Service Data or hardcopy tool
2	Between 12:00 AM and 11:59 PM	Unstructured Supplementary Service Data or hardcopy tool
3	Between 12:00 AM and 11:59 PM	Unstructured Supplementary Service Data or hardcopy tool
4	Between 12:00 AM and 11:59 PM	Unstructured Supplementary Service Data or hardcopy tool
5	Between 12:00 AM and 11:59 PM	Unstructured Supplementary Service Data or hardcopy tool
6	Between 12:00 AM and 11:59 PM	Unstructured Supplementary Service Data or hardcopy tool
7^b^	Between 12:00 AM and 11:59 PM	Unstructured Supplementary Service Data or hardcopy tool

^a^Day of vaccination.

^b^New or unresolved reactogenicity symptoms present on day 3 are followed until resolution.

Participants allocated to the USSD arm are instructed to await a reminder text message daily during the 7-day reactogenicity period to access the system, enter the unique code, and complete the tool. Any symptoms reported and graded subjectively by the participant as moderate result in an immediate alert to selected research staff, via SMS text message to their mobile phones. A clinical staff member will then contact the participant by telephone to determine whether the objective grading, as per the DAIDS adult and pediatric toxicity table [18], is confirmed. If it is confirmed, the file is flagged to check the grading the next day on the database to determine whether a clinic visit is required, as per the protocol. Alerts for unexpected symptoms will also result in a site-initiated telephone contact to obtain detail, grade the symptom objectively, and facilitate reporting. Printouts from the USSD database of completed reactogenicity assessments per day will be stored in the participant binder following transcription onto a CRF and entry into the parent study database. Participants complete the USSD tool daily for 8 days on the day and at the time of the assessment; no phone calls from the site are necessary unless grade 2 or higher symptoms are reported, repeated entries are made, or the system shows that the participant has not logged in by 15:00 of the same day. If a participant misses a day, they can complete the tool retroactively for the previous day. If symptoms increase in severity after the USSD tool has been completed, it may be updated by the participant on the same day. Any dual entries on a single day prompt a call from site staff to ascertain the reason, which is documented in the site records.

Symptoms that are present at day 7 can only be detected contemporaneously and followed up in the intervention arm, allowing follow-up until resolution at a frequency determined by the investigator, for example, a call a few days later to document a resolution date. For the hardcopy tool, symptoms present at day 7 will only be known at the follow-up safety visit when the hardcopy tool is returned and reviewed or if the participant proactively alerts the site staff. If the participant does not document this in the hardcopy tool on the day that symptoms resolve, recall bias may affect the accuracy of the data when checked at the safety visit.

A backup hardcopy tool is given to all participants assigned to the USSD arm, so that in the event of technical challenges that cannot be resolved by site or provider staff, reactogenicity data are not lost to the parent study.

#### Termination From the Study

Under certain circumstances, an individual participant may be terminated from participation in this study. Specific events that will result in early termination are (1) the participant refuses further participation, (2) the participant no longer possesses a mobile phone, (3) the participant is terminated from the parent study before the vaccination series is completed, (4) the participant relocates, (5) research staff determine that the participant is lost to follow-up, (6) the investigator decides in consultation with the coinvestigator to terminate participation, for example, if a participant exhibits inappropriate behavior toward clinic staff, and (7) any condition where termination from the study is required by applicable regulations.

In the event of early participant termination from the ancillary study only, research staff would consider whether the following assessments are appropriate: a final feasibility and acceptability questionnaire with participant consent, and a reminder in the participant binder to provide a hardcopy tool at the next visit, if appropriate. Data already collected would still be used in the final analysis.

The pMOTAR may be terminated early by the determination of the parent protocol Safety Review Team, the regulatory body, the ethics committee, or the sponsor.

#### Social Impacts

Social impacts occur when participants experience any psychological, legal, economic, or emotional harms as a direct result of their participation in a research study; these will be reported in the parent study. The possibility of participants in this ancillary study experiencing social impacts is low to none, as data are deleted from the device automatically on submission and can only be accessed with the unique 4-digit identifying code.

## Results

The USSD app has been developed and implemented. Data collection has been completed, and results will be published in a primary paper.

## Discussion

### Implications of the Research

If collection of reactogenicity data by the USSD tool is proven to be feasible, accessible, and superior in completeness and accuracy to the hardcopy tool, it will leverage the use of mobile phones and USSD apps for routine collection of reactogenicity data in future safety and efficacy studies of vaccine candidates, and in eventual programmatic rollout. Our experience in development and implementation of the USSD app will allow for adjustments to any future development and implementation in order to reduce challenges and facilitate seamless collection of data across all sites and studies, with appropriate training. If successful, it will facilitate contemporaneous reactogenicity data capture, while improving efficiency and accuracy. Accurate safety data collection may allay participant and public fears of side effects, leading to increased uptake of HIV vaccines in research studies, and eventually in programmatic rollout. Improved uptake will contribute to producing the herd immunity required to ensure a halt to transmission in endemic areas.

### Strengths and Limitations

Using a randomized controlled clinical trial to assess feasibility, accessibility, completeness, and accuracy of the USSD is a key strength of this study. Ensuring that the USSD and hardcopy tools collect exactly the same safety data means that the safety end points and potential impact on the objectives and analysis of the parent study are not compromised. Unlike the hardcopy tool, the USSD tool cannot be lost, even if the mobile phone is lost, because the data having been uploaded to the database will still be available for entry onto CRFs. From a technology innovation perspective, the selection of USSD as the delivery mechanism for the intervention is an added strength, based on accessibility on any basic mobile phone. Contemporaneous alerts to research staff of moderate symptoms allows for real-time follow-up to ensure symptoms are resolving and to assess whether a clinic assessment is required or a parent study pause rule has been met. Erroneous temperature data can also be immediately identified, and the participant can be contacted in a timely manner to repeat the measurement. The USSD unique code increases the likelihood that the participant is completing the data; the prepopulation of the user, dates, and times recorded by the system on log-in removes duplication of data fields for possible error, thus improving the overall quality of the data. Data can be accessed in real time by staff and transcribed onto CRFs, facilitating earlier entry into the parent study database. Confidentiality is assured by autodeletion of any data entries after completion of a USSD string and submission to the tool database, averting the possibility of social harm. Preservation of the last active screen builds in efficiency, so that a participant does not double enter data for the same data field erroneously.

Limitations include that the study is not blinded, in terms of allocation of intervention; however, data analysis staff at the Statistical Data Management Centre will be blinded to the allocation at the time of analysis of the parent study safety data, thus maintaining the integrity of the parent study blinding. Errors in USSD entry cannot be corrected in real time without a duplicate entry on the same or next day. The USSD tool is limited to text only, and the lack of graphical representations, including the use of bold typeface and color, reduces the attractiveness of the user interface, as well as the appeal to participants who respond to graphics more easily than text. Technological challenges when the network signal is poor or handsets are not fully charged are also potential limitations, as is the recruitment from only 1 site and the small sample size, which reduces the generalizability of this study and the reliability of the answer. It is possible that countries with lower mobile phone coverage and access may have reduced text literacy, which would affect the utility of the tool across regions.

### Conclusion

This is, to our knowledge, the first South African randomized clinical trial to test the feasibility and accessibility of the USSD tool for collection of reactogenicity data in an HIV vaccine trial, and to attempt to prove superior accuracy and completion to the hardcopy tool. This would, we hope, improve the reliability of safety data and possibly increase uptake of vaccines through accurate reporting of safety data.

## Appendix

Multimedia Appendix 1 Survey for participants on acceptability of the pilot study of Mobile Technology to Assess Reactogenicity (pMOTAR) app.

Multimedia Appendix 2 Survey for participants on acceptability of the hardcopy tool.

Multimedia Appendix 3 Survey for staff members on acceptability of the pilot study of Mobile Technology to Assess Reactogenicity (pMOTAR) app.

## Abbreviations

CAPRISA: Centre for the AIDS Programme of Research in South Africa
CRF: case report form
DAIDS: Division of AIDS
ECRS: eThekwini Clinical Research Site
HVTN: HIV Vaccine Trials Network
mHealth: mobile health
SMS: short message service
USSD: Unstructured Supplementary Service Data
