# Supplementary figures and images for: Using Mobile Technology (pMOTAR) to Assess Reactogenicity: Protocol for a Pilot Randomized Controlled Trial
Source: JMIR Res Protoc. 2018 Oct 3;7(10):e175. doi: 10.2196/resprot.9396 (PMC6231778; doi:10.2196/resprot.9396)

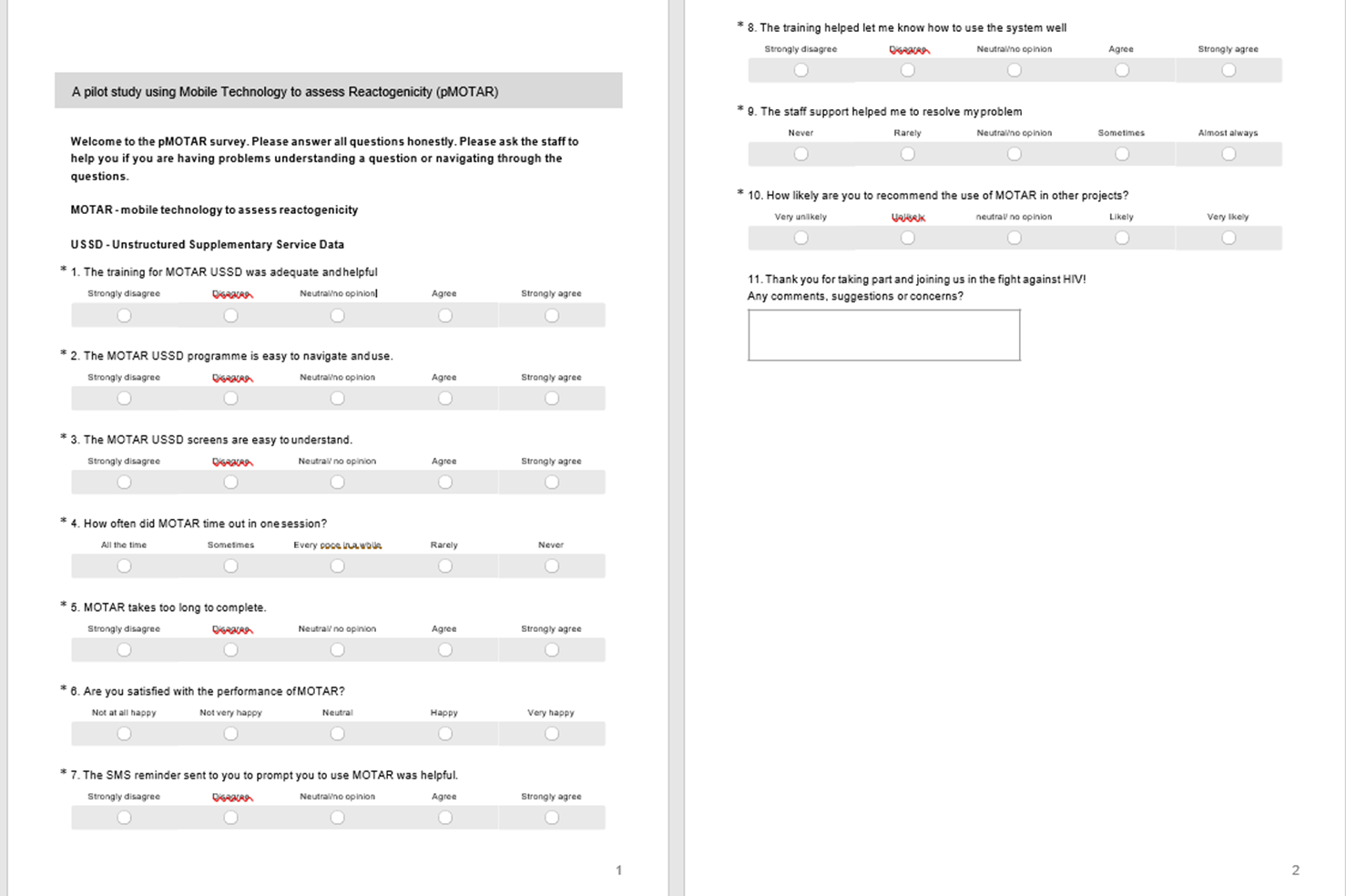

Supplement: Multimedia Appendix 1 [file resprot_v7i10e175_app1.png]

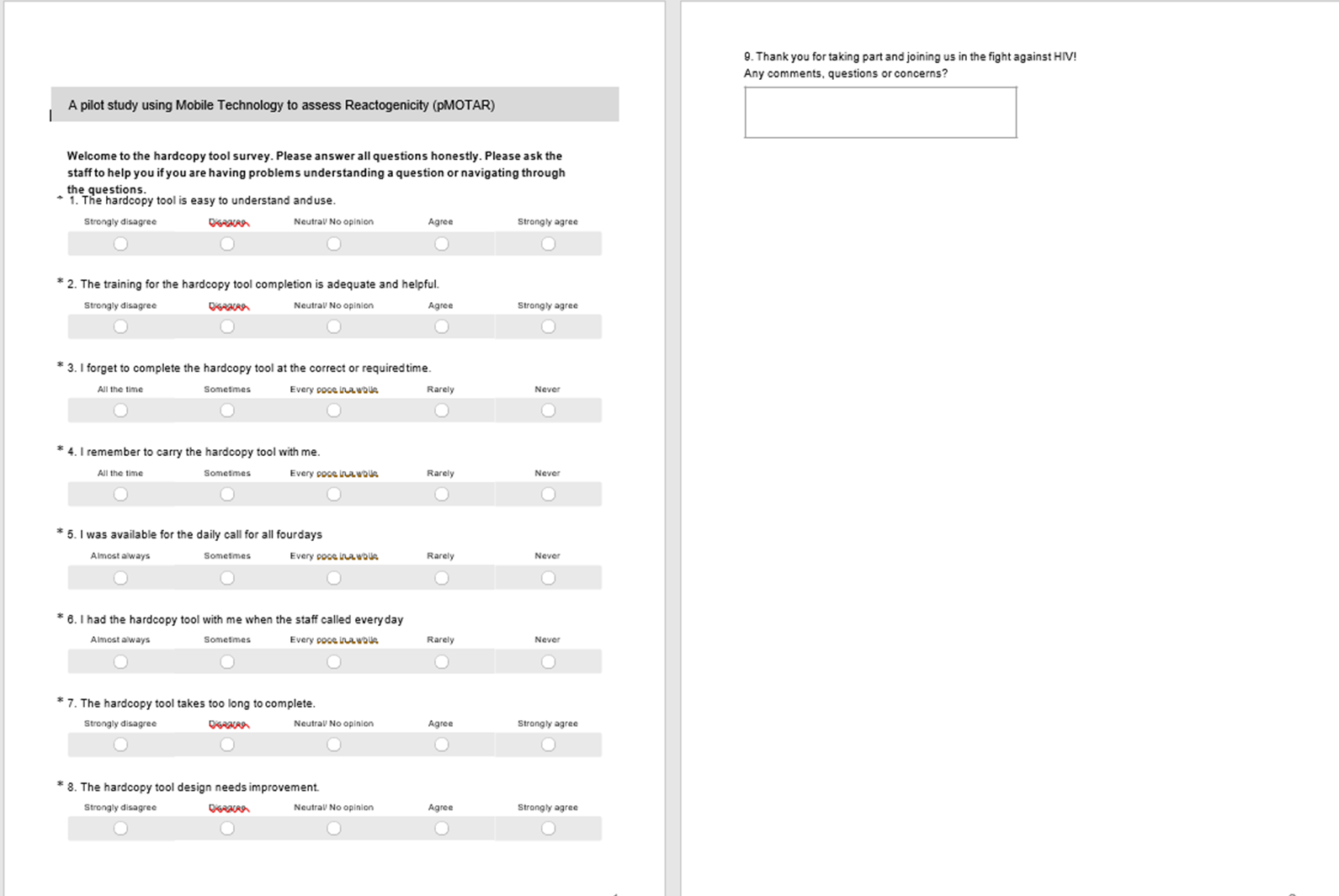

Supplement: Multimedia Appendix 2 [file resprot_v7i10e175_app2.png]

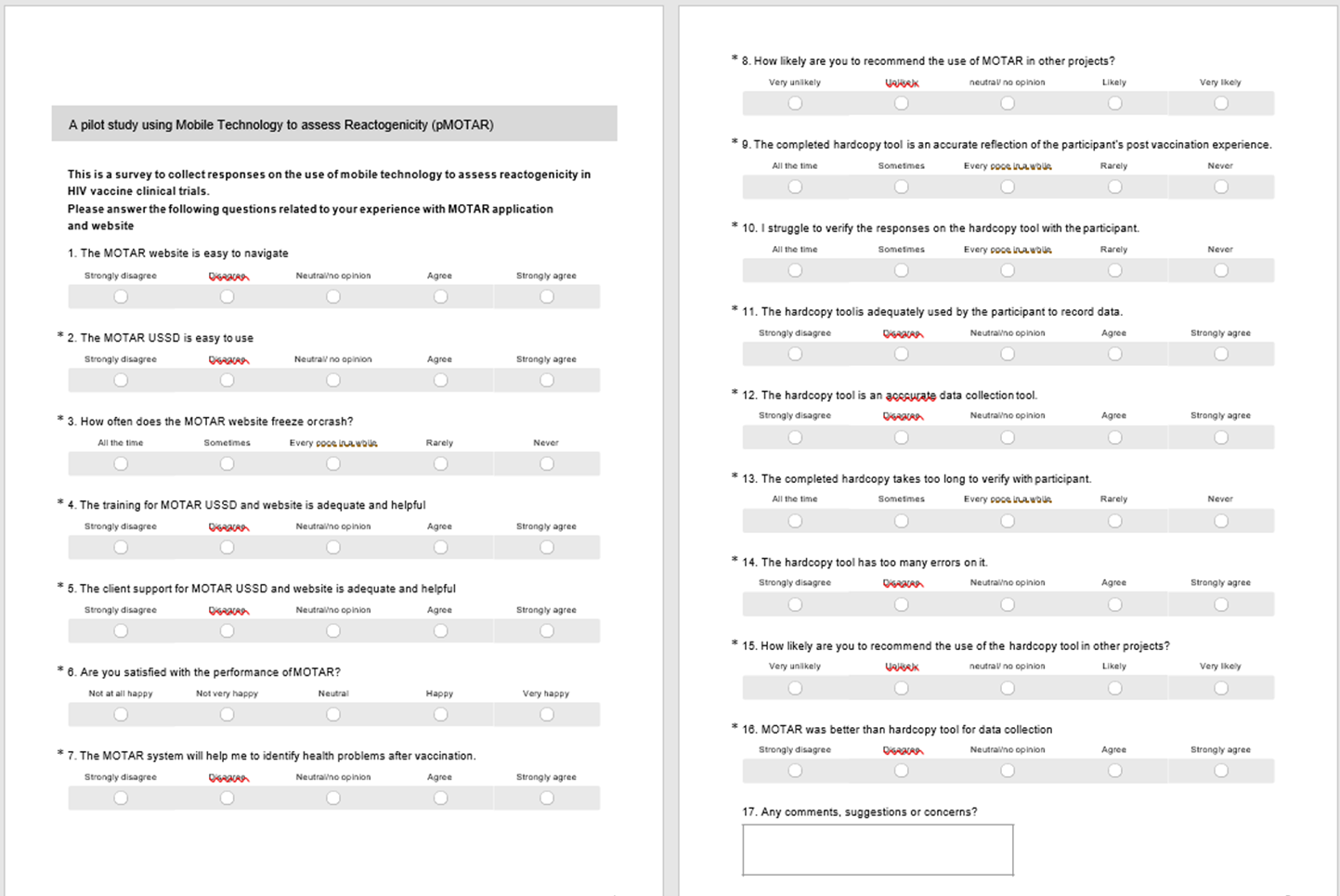

Supplement: Multimedia Appendix 3 [file resprot_v7i10e175_app3.png]
